# Image recognition technology for bituminous concrete reservoir panel cracks based on deep learning

**DOI:** 10.1371/journal.pone.0318550

**Published:** 2025-02-04

**Authors:** Kai Hu, Yang Ling, Jie Liu

**Affiliations:** 1 School of Civil Engineering and Architecture, Xi’an Technological University, Xi ’an, Shaanxi, China; 2 Shaanxi Qinyuan Tendering Co., Ltd, Xi ’an, Shaanxi, China; 3 Shanxi Yuanqu Pumped Storage Co., Ltd, Yuncheng, Shanxi, China; Oregon State University, UNITED STATES OF AMERICA

## Abstract

Detecting cracks in asphalt concrete slabs is challenging due to environmental factors like lighting changes, surface reflections, and weather conditions, which affect image quality and crack detection accuracy. This study introduces a novel deep learning-based anomaly model for effective crack detection. A large dataset of panel images was collected and processed using denoising, standardization, and data augmentation techniques, with crack areas labeled via LabelImg software. The core model is an improved Xception network, enhanced with an adaptive activation function, dynamic attention mechanism, and multi-level residual connections. These innovations optimize feature extraction, enhance feature weighting, and improve information transmission, significantly boosting accuracy and robustness. The improved model achieves a 97.6% accuracy and a Matthews correlation coefficient of 0.98, remaining stable under varying lighting conditions. This method not only provides a fresh approach to crack detection but also greatly enhances detection efficiency.

## 1. Introduction

As an important part of the water conservancy project, the safety of asphalt concrete reservoir [1, 2] is directly related to the operation stability of the reservoir and the safety of surrounding areas. Faceplate crack is a common key problem in reservoir structure, which is caused by various factors, such as material aging, environmental impact and seismic activity. The components of the asphalt panel dam include the panel, cushion layer, dam body and downstream drainage body. The asphalt concrete panel is a mixture of asphalt, mineral powder, sand, stone chips and small gravel heated to about 220 degrees, evenly spread on the upstream dam surface after mixing, and compacted at about 180 degrees. The asphalt concrete panel has good anti-seepage and deformation adaptability, and has certain water stability and thermal stability. If these fractures cannot be found and solved in time, it may endanger the stability of the reservoir and may lead to disastrous consequences. Therefore, it is very important to accurately and timely identify and monitor panel cracks. Traditional crack detection methods rely on manual inspection and analysis to a great extent, which is time-consuming and laborious, and prone to human error, thus limiting the detection accuracy. These methods usually need experienced professionals, using visual inspection, acoustic testing, ultrasonic testing and other technical means to evaluate. However, these technical means are limited by the detection environment and human factors, so it is difficult to fully cover all cracks in the reservoir panel [3, 4], and the efficiency is low. By training the depth neural network model, the automatic recognition of crack images can be realized, thus significantly improving the accuracy and efficiency.

The method of deep learning [5, 6] has made a breakthrough in the field of image recognition. Convolutional neural networks [7, 8], generating countermeasure networks and other models have performed well in image classification, object detection and image segmentation. Specifically in the field of reservoir panel crack detection, the enhanced anomaly model based on deep learning [9, 10] can further improve the accuracy and reliability of detection. Alhebrawi Mohamad Najib [11] proposed an automatic crack recognition process for impact hammer testing. Based on the fast Fourier transform of hammering sound, three methods were used to recognize crack features, such as width, depth, and position. It was confirmed that the support vector machine algorithm had the ability and effectiveness to accurately recognize concrete fine cracks with a width of 0.2mm and a depth of 40mm. Yang Qun [12] proposed a transverse crack recognition method based on vehicle vibration using a support vector machine. The support vector machine maximized the classification boundary for classification, which made it perform well in high-dimensional spaces, accurately recognizing crack features, and mapping data to higher-dimensional spaces through kernel functions to achieve more accurate classification. However, these methods have limitations in feature extraction and pattern recognition, and cannot effectively solve the complex problems in crack recognition. Lack of effective extraction and recognition methods for crack image features results in low recognition accuracy and sensitivity to environmental factors.

Convolutional neural networks and transfer learning have been used by certain researchers, with some success [13, 14]. Convolutional neural networks [15, 16] eliminate the laborious human feature engineering seen in conventional techniques by automatically extracting hierarchical characteristics of pictures using convolutional and pooling layers. Because of its multi-layer structure, fracture details and complicated morphology may be captured. By developing appropriate models, convolutional neural networks can identify fractures in a variety of materials, including metal, concrete, and other materials. A two-step convolutional neural network-based technique for detecting and segmenting road cracks was presented by Liu Jingwei [17]. The updated automated road fracture detecting technique was applied in the first stage. The second step’s suggested crack segmentation approach was based on an enhanced U-Net model, and it demonstrated an accuracy advantage over the current one-step road fracture detection or segmentation methods. Using a novel multi-scale convolutional feature fusion module, Qu Zhong [18] introduced a deep supervised convolutional neural network for crack identification. High-level features were directly applied to low-level features of various convolution levels in this multi-scale feature fusion module. These techniques have a high degree of generalization capacity and can automatically identify features in pictures; nevertheless, there are still problems with sensitivity to various environmental conditions and the need to increase crack detection accuracy.

In response to the above issues and challenges, to further improve the accuracy and stability of crack image detection in bituminous concrete reservoir panels, the Xception deep convolutional neural network model was improved from multiple directions based on deep learning technology. The image data of bituminous concrete reservoir panel was extensively collected, including four categories: transverse cracks, longitudinal cracks, massive cracks, and normal cracks, and the training dataset was further expanded through data augmentation techniques. Based on the Xception deep model, the attention mechanism and residual connection were integrated to improve the recognition accuracy and the contrast performance of crack images. The Improved Model 7 in this article demonstrates excellent performance and significantly improves the accuracy and robustness of crack detection. By optimizing the network structure and adjusting hyperparameters, the model shows higher sensitivity and accuracy when dealing with complex backgrounds and tiny cracks, enhancing the recognition effect in practical applications. This study uses deep learning technology to accurately identify asphalt concrete images and innovatively apply it to reservoir panel crack detection. The convolutional neural network is used to achieve high-precision crack feature extraction and classification, which greatly improves the accuracy and efficiency of crack identification. This method effectively solves the problems of false detection and missed detection in traditional detection methods, and provides an intelligent and automated solution for reservoir maintenance. This article proposes an asphalt concrete image recognition technology based on deep learning, especially for the automatic detection of reservoir panel cracks. By combining convolutional neural networks and advanced data augmentation techniques, the accuracy and robustness of crack identification are significantly improved. By introducing an adaptive learning rate strategy, the model’s performance in complex environments is further improved, providing an efficient solution for asphalt concrete structural health monitoring.

## 2. Materials and methods

### 2.1 Collection of crack image data

By leveraging the technology of automated crack recognition [19, 20], it becomes possible to swiftly and precisely identify cracks present on panels of reservoirs. This capability offers a proactive alert system for personnel responsible for reservoir management, thus averting the escalation of crack-related issues that could potentially cause severe structural complications. The data pertaining to crack images captured from diverse reservoir panels encompasses a wide array of crack types and is obtained under varying environmental circumstances, encompassing alterations in lighting conditions and shooting perspectives.

Cracks are generally not obvious enough, and light conditions are very important for crack image recognition. Image data in three types of environments is collected: dark light, normal light, and bright light. The light intensity between 10 and 300 lux is set as dark light; the light intensity between 300 and 800 lux is set as normal light; the light intensity between 800 and 10000 lux is set as bright light.

**Fig 1:**
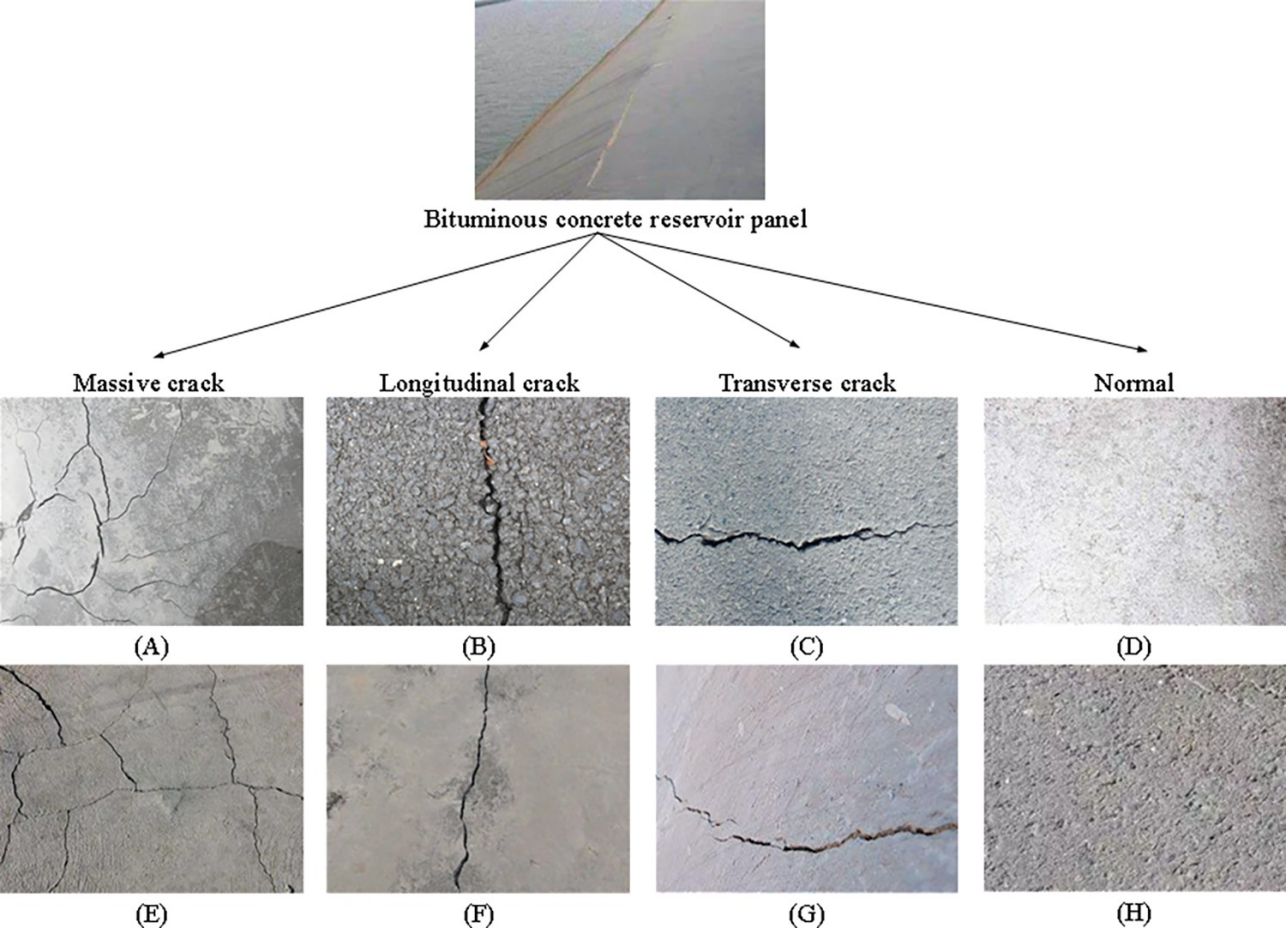
Image of bituminous concrete reservoir panel.

High-resolution digital cameras are used to capture detailed information of cracks in three types of lighting environments. The new dataset contains images from multiple geographic regions and environmental conditions, covering different seasonal changes and multiple types of reservoirs. The data was collected from June to December 2023 and captured using a high-resolution digital camera (Canon EOS 5D Mark IV) to ensure clear presentation of crack details. The image data comes from the Xiaolangdi Reservoir of the Yellow River in Henan Province, China. A variety of climate, light and environmental factors are taken into account, which helps to improve the universality and robustness of the crack detection model. Since the collected image data is not much, the crack image dataset in this paper was constructed by combining the images in the public dataset rdd2022. The collected image information is shown in Fig 1.

800 original bituminous concrete reservoir panel images are collected in three types of environments: dark light, normal light, and bright light. A dataset of bituminous concrete reservoir panel images for three different environments is established. By classifying and recognizing these four types of images, the crack situation of the reservoir structure can be effectively monitored; crack problems can be detected in a timely manner; corresponding repair measures can be taken to ensure the safe operation and structural stability of the reservoir.

### 2.2 Preprocessing and data annotation

#### (1) Preprocessing

During the image acquisition process, various noises may exist in the image due to environmental factors, equipment conditions, and other factors. Preprocessing the collected images is to improve the performance and accuracy of the algorithm in the process of crack recognition and analysis. To adapt to input images of different sizes, the images are adjusted to a uniform 1920x1080 pixel size. The image data is preprocessed, including denoising [21, 22], normalization, data augmentation [23, 24], and other processes.

The formula for denoising processing is expressed as:

$$I_{new}(x,y)=median(I_{nei}(x,y))$$ (1)

De-noising is based on the median filter algorithm, which effectively removes noise from the image by replacing the current pixel value with the median value in the pixel neighborhood. Median filtering is particularly suitable for salt and pepper noise because it can preserve edge information and effectively suppress the impact of noise on image quality. Compared with mean filtering, median filtering does not blur image edges, can better preserve image details, and improve the accuracy and robustness of subsequent processing.

The formula for image normalization is expressed as:

$$I_{g}=\frac{I-I_{min}}{I_{max}-I_{min}}$$ (2)

#### (2) Data augmentation

**Fig 2:**
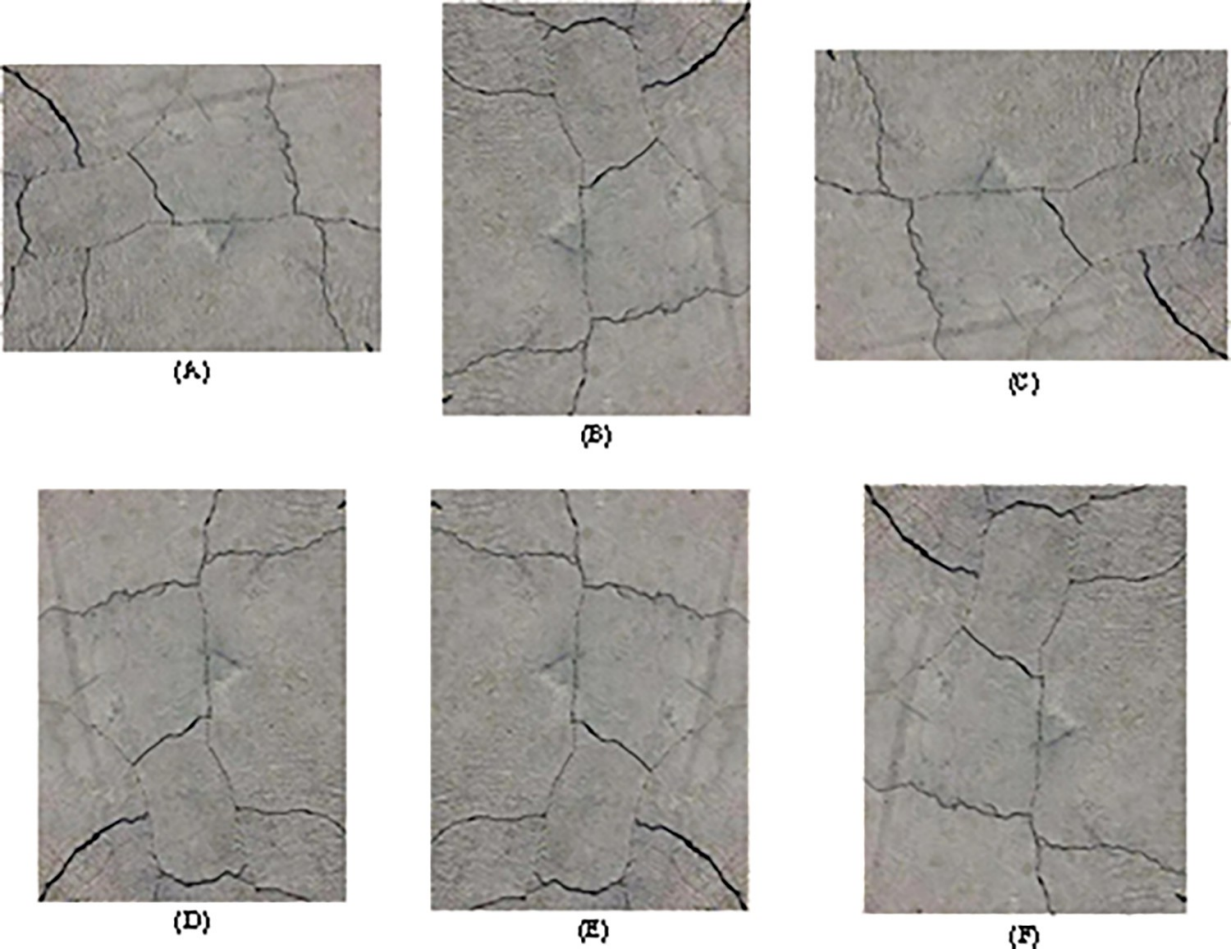
Effect of image expansion.

The collected raw image dataset is subjected to data augmentation, which increases data diversity and improves the model’s generalization ability through operations such as rotation and flipping. The effect of image expansion is shown in Fig 2.

In Fig 2(A)–2(F), they represent the original image, rotated 90° to the right, 180° to the right, 270° to the right, horizontally flipped, and vertically flipped images, respectively. Through data augmentation processing, the number of images is expanded to six times the original number of images. Data augmentation can apply different changes and noise, and simulate diversity and complexity in the real world, making the model more robust and better able to adapt to different scenarios and environmental changes. Data augmentation technology is used to increase the diversity and quantity of training data, thereby improving the generalization ability of the model. By rotating, flipping, cropping and other transformations on the original image, the environmental changes and uncertainties in the real world are simulated to help the model better adapt to different scenarios and conditions. This method can effectively prevent overfitting and improve the model’s ability to recognize unknown data. In addition, data augmentation can also balance the imbalance of different categories of data and improve classification accuracy and robustness, which is particularly important in practical applications such as crack detection.

Through data augmentation, there are 4800 images in each type of dataset. The dataset is divided in a 3:1 format. The image dataset with normal light, dark light, and bright light is shown in Tables 1–3.

**Table 1:** Image dataset under normal light.

Type	Original image	Expanded image	Training set	Test set
Transverse crack	222	1332	999	333
Longitudinal crack	200	1200	900	300
Massive crack	200	1200	900	300
Normal	178	1068	801	267
Total	800	4800	3600	1200

**Table 2:** Image dataset with dark light.

Type	Original image	Expanded image	Training set	Test set
Transverse crack	210	1260	945	315
Longitudinal crack	190	1140	855	285
Massive crack	190	1140	855	285
Normal	210	1260	945	315
Total	800	4800	3600	1200

**Table 3:** Image dataset with bright light.

Type	Original image	Expanded image	Training set	Test set
Transverse crack	208	1248	936	312
Longitudinal crack	212	1272	954	318
Massive crack	210	1260	945	315
Normal	170	1020	765	255
Total	800	4800	3600	1200

#### (3) Image annotation

**Fig 3:**
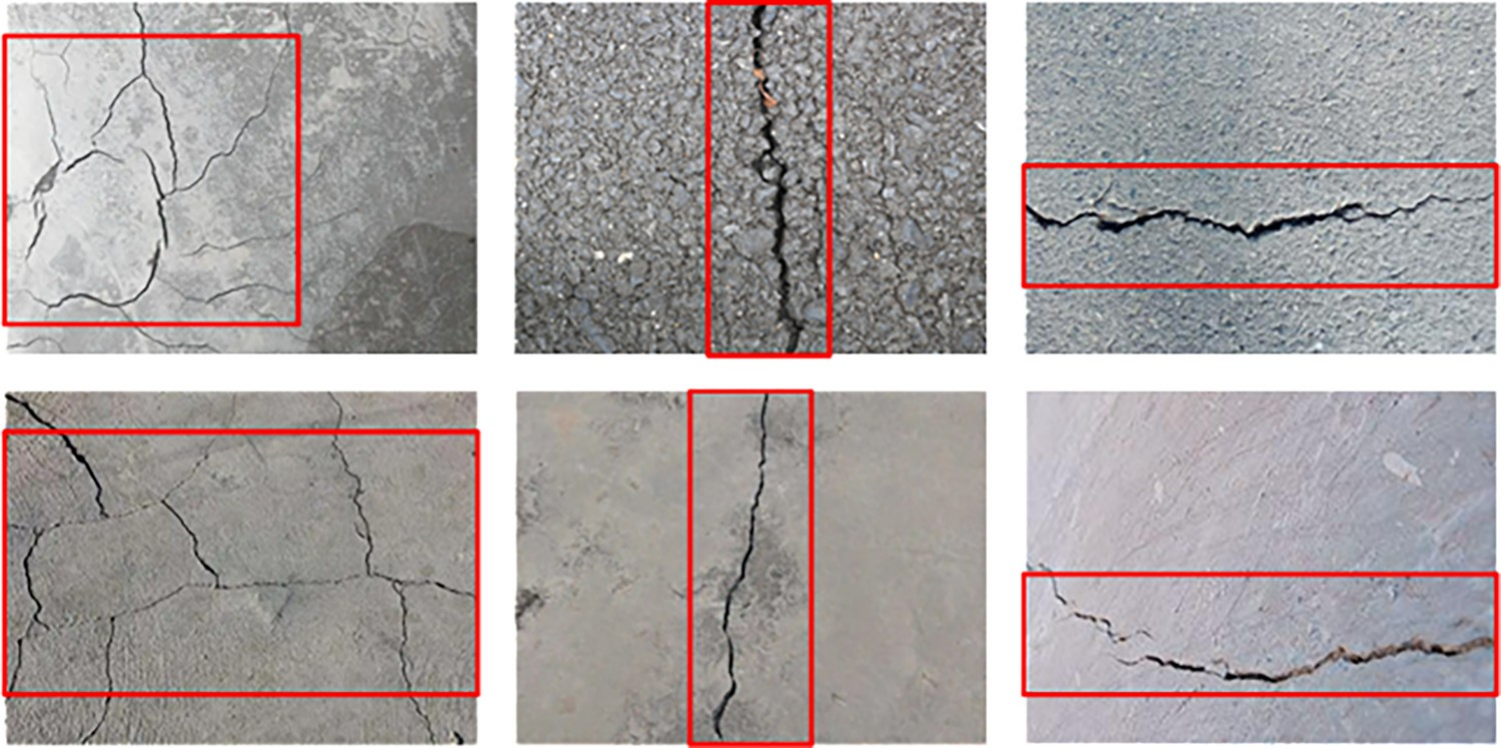
Results of crack area annotation.

LabelImg annotation software is used to annotate crack areas in images. The results of crack area annotation are shown in Fig 3.

The images that need to be marked are imported, and the crack areas in the images are selected through a red box. Each image is annotated with the content of Massive crack, Longitudinal crack, Transverse crack, Normal. Accurate annotation can help the model better learn the characteristics of cracks, and distinguish different types of cracks and normal areas.

### 2.3 Xception model improvement

The Xception model [25, 26] is a deep convolutional neural network, and it is selected as the base model. Xception is a convolutional neural network based on depthwise separable convolution, which has strong feature extraction ability and less computational complexity. Although the Xception model performs well in image recognition and classification tasks, it still has some shortcomings.

The formula for ReLU is:

ReLU=max(0,x) (3)

When the input of ReLU [27, 28] is negative, its output is zero. If the input of a large number of neurons remains negative, these neurons would never be activated, causing them to no longer update during the training process, thereby affecting the learning ability of the model.

In order to improve the image recognition effect of bituminous concrete reservoir panel cracks, this article improves the Xception model from three directions. The improvements include improving the activation function, applying attention mechanism, and adding residual connections [29, 30].

**Fig 4:**
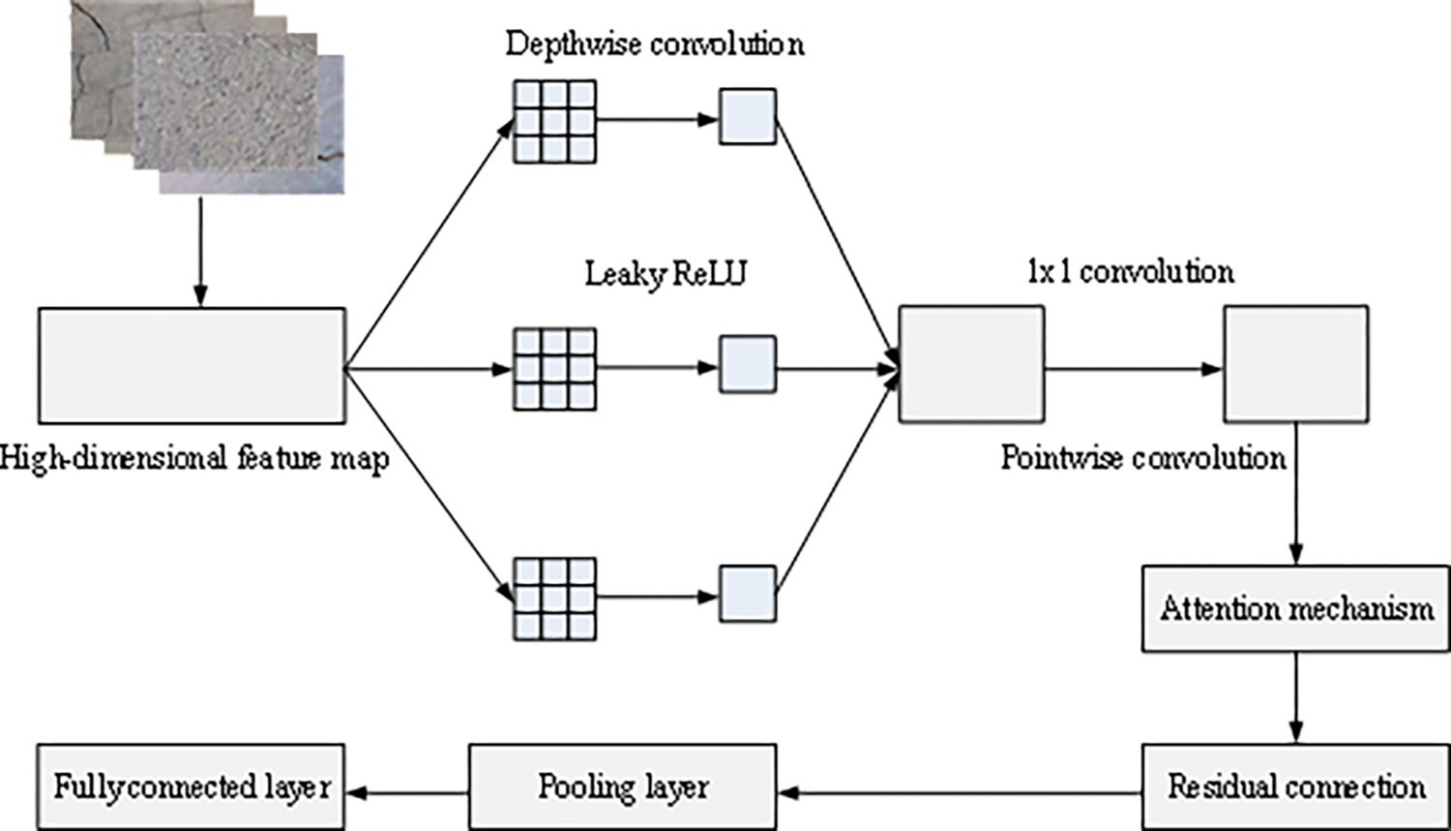
Improved Xception model structure.

The improved Xception model structure is shown in Fig 4.

In Fig 4, Leaky ReLU is used as the activation function to improve the training performance and non-linear expression ability of the model. In order to enhance the model’s attention to crack areas, attention mechanism is applied, which automatically adjusts the weight of the model to different image regions and improves the ability to capture key features. The attention mechanism assigns different weights to input features, allowing the model to focus more on important parts and improve its ability to capture key features. The global features of each channel are calculated, and attention weights are generated through a two-layer fully connected network. The weights are normalized to [0,1] using the sigmoid function. The attention weight is readjusted to the same dimension as X, *C*×*H*×*W*, and then multiplied with the original feature map X to obtain the weighted feature map *X*′. In the model, residual connections are applied to directly transmit input information to subsequent layers through skip connections. The input feature is denoted as x, and the feature F(x) is calculated through several convolutional layers. The input x is added to F(x) to obtain the output y. The output y of the residual block is passed to subsequent layers for further processing. Residual connections can effectively alleviate the problems of vanishing and exploding gradients, improve the training effectiveness and stability of deep networks, and accelerate convergence.

Leaky ReLU applies a small negative slope to prevent neuronal death. The formula is expressed as:

$$f(x)=\left\{ \begin{array}{c} x,\;if\;x>0\\ ax,\;if\;x\leq 0 \end{array}\right.$$ (4)

In each convolutional module, a skip connection is added to directly add the input to the output. By improving the activation function and applying attention mechanism and residual connections, the improved Xception model can pay more attention to crack areas and perform well in crack detection tasks.

## 3. Experimental conditions and evaluation

This article improves the Xception model to achieve crack image recognition. The processing of images and data is carried out through OpenCV 4.5.2 and Pandas 1.2.4. The model in this article includes the following network parameters: the convolution layer has 32 filters of size 3x3 and stride 1; the maximum pooling layer size is 2x2; the fully connected layer contains 128 neurons; the activation function is ReLU; the output layer uses the softmax activation function for classification. The optimizer uses Adam, and the learning rate is set to 0.001. The improved Xception model extracts image features through multiple convolutional layers and pooling layers to enhance the recognition ability of the model. The fully connected layer and the softmax output layer ensure high-precision classification results, and the Adam optimizer improves the stability and efficiency of training.

Pavement cracks are usually classified according to their morphology, causes, and development characteristics. Common crack types include transverse cracks, longitudinal cracks, oblique cracks, and block cracks. Transverse cracks are generally distributed horizontally and are often caused by temperature changes or loads. They usually appear at the joints of road panels [31, 32]. Longitudinal cracks extend along the length of the pavement and are common in stress concentration areas caused by uneven foundation settlement or material aging. Oblique cracks are usually at a 45-degree angle and are usually caused by uneven settlement or construction quality problems. Block cracks usually appear as large area damage, forming blocky or cracked structures, which are common in material fatigue caused by pavement overload or long-term use. The type of crack directly affects the structural strength and service life of the road. Therefore, accurate identification and classification of crack types is the key to effective pavement maintenance and management [33, 34]. With the advancement of technology, crack classification methods based on image recognition and deep learning can automatically extract crack features, improve detection efficiency and accuracy, and provide a reliable basis for pavement maintenance.

The loss function formula is expressed as:

$$L(y,\hat{y})=-(ylog(\hat{y})+(1-y)log(1-\hat{y}))$$ (5)

This article improves the Xception model in three directions: setting the activation function to Leaky ReLU, applying attention mechanism, and adding residual connections. ResNet50, Inception, GoogLeNet, and improved Xception are compared under normal light conditions in order to demonstrate the recognition effectiveness of the improved Xception model.

The recognition performance is evaluated from different directions through recognition accuracy, Matthews correlation coefficient, and Receiver Operating Characteristic (ROC). The four categories of classification problems are transformed into binary classification problems, where each binary classification problem takes one category as a positive example and the other categories as negative examples. The formula for calculating the accuracy of a certain category classification is:

$$Acc=\frac{TP+TN}{TP+TN+FP+FN}$$ (6)

The formula for the Matthews correlation coefficient of a certain category classification is:

$$MCC=\frac{TP\times TN-FP\times FN}{\sqrt{\left(TP+FP)(TP+FN)(TN+FP)(TN+FN\right)}}$$ (7)

The ROC curve provides a comprehensive perspective, demonstrating the performance of the model under different decision thresholds, and can help understand the effectiveness of the model under different trade-offs of true positive and false positive rates. By comparing the ROC curves of different models, it can be visually determined which model performs better in distinguishing positive and negative samples.

The model information of the ablation experiment is shown in Table 4.

**Table 4:** Model information for ablation experiments.

Model	Leaky ReLU	Attention mechanism	Residual connection
Xception	-	-	-
Improved model 1	√	-	-
Improved model 2	-	√	-
Improved model 3	-	-	√
Improved model 4	√	√	-
Improved model 5	√	-	√
Improved model 6	-	√	√
Improved model 7	√	√	√

(Note: √ represents the improvement content adopted; - represents the improvement content not adopted; Improved model 7 is the improved Xception model adopted in this article)

In Table 4, the model information of the ablation experiment is presented, and the Xception model is compared with six improved models. The optimal improved model can be determined through ablation experiments, which can effectively improve the image recognition effect of cracks in bituminous concrete reservoir panels.

In practical applications, the lighting environment changes with time and seasons, so the stability of the model is crucial for the reliability of crack image recognition. This article conducts image recognition verification in three types of environments: dark light, normal light, and bright light, and analyzes the stability of the model.

## 4. Results

### 4.1 Results of ablation experiments

**Fig 5:**
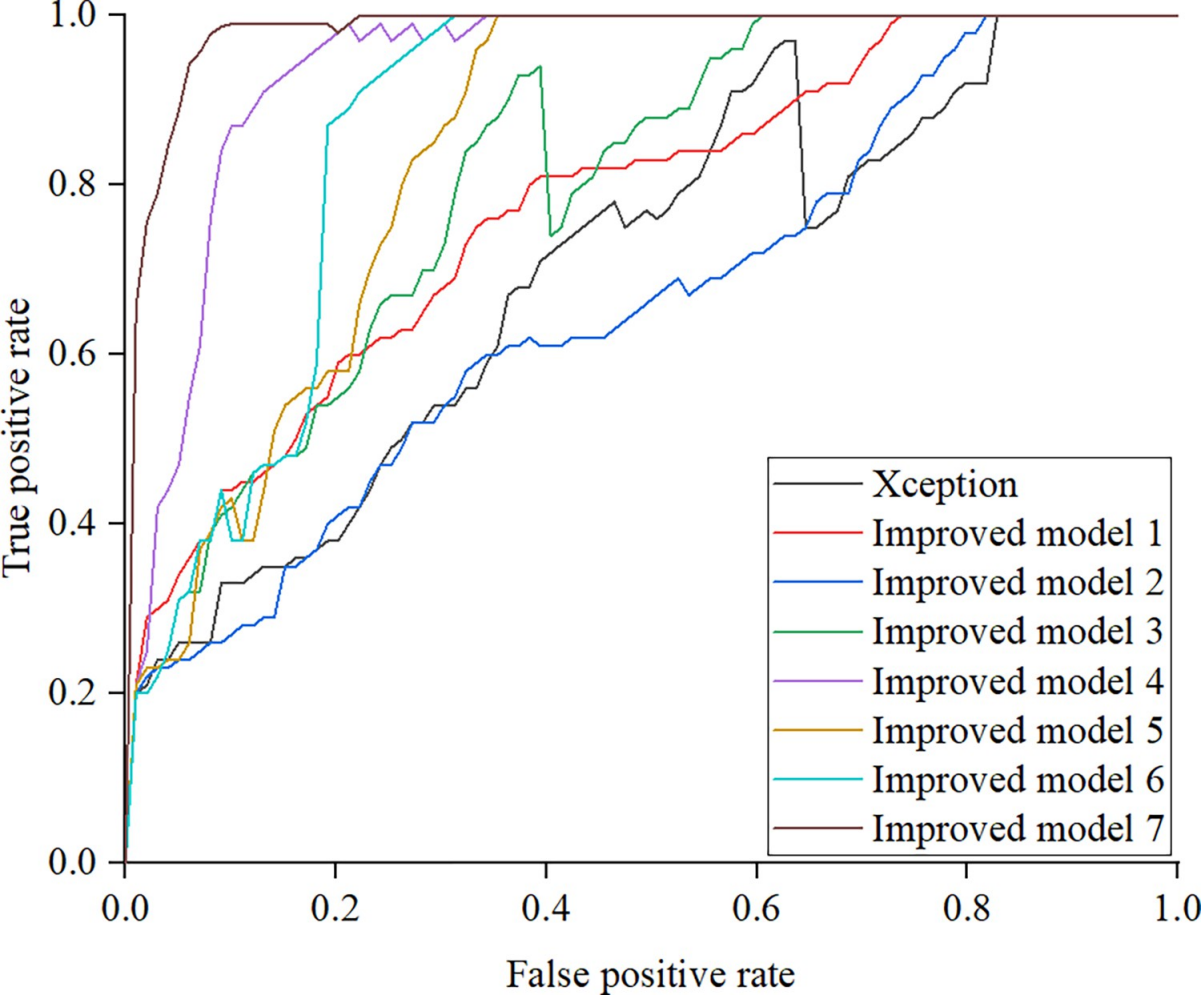
Results of ablation experiment.

There are a total of 8 models participating in the ablation experiment. Under normal light conditions, the ROC comparison results of the models participating in the ablation experiment are shown in Fig 5.

This article improves the Xception model in three directions: improving the activation function, applying attention mechanism, and adding residual connections. Seven improved Xception models are generated based on the combination of improved content. Improved model 7 has a larger area under the curve. The application of activation functions avoids the problem of “death meridian elements”, making the model perform better in handling negative information. The attention mechanism enables the model to better focus on the key areas of cracks, improving the pertinence and effectiveness of feature extraction. By optimizing the residual connection structure, information can be smoothly transmitted in deep networks, alleviating the problems of gradient vanishing and exploding. Therefore, the improved model used in this article is Improved Model 7, which has been improved in three directions.

### 4.2 Recognition accuracy

**Fig 6:**
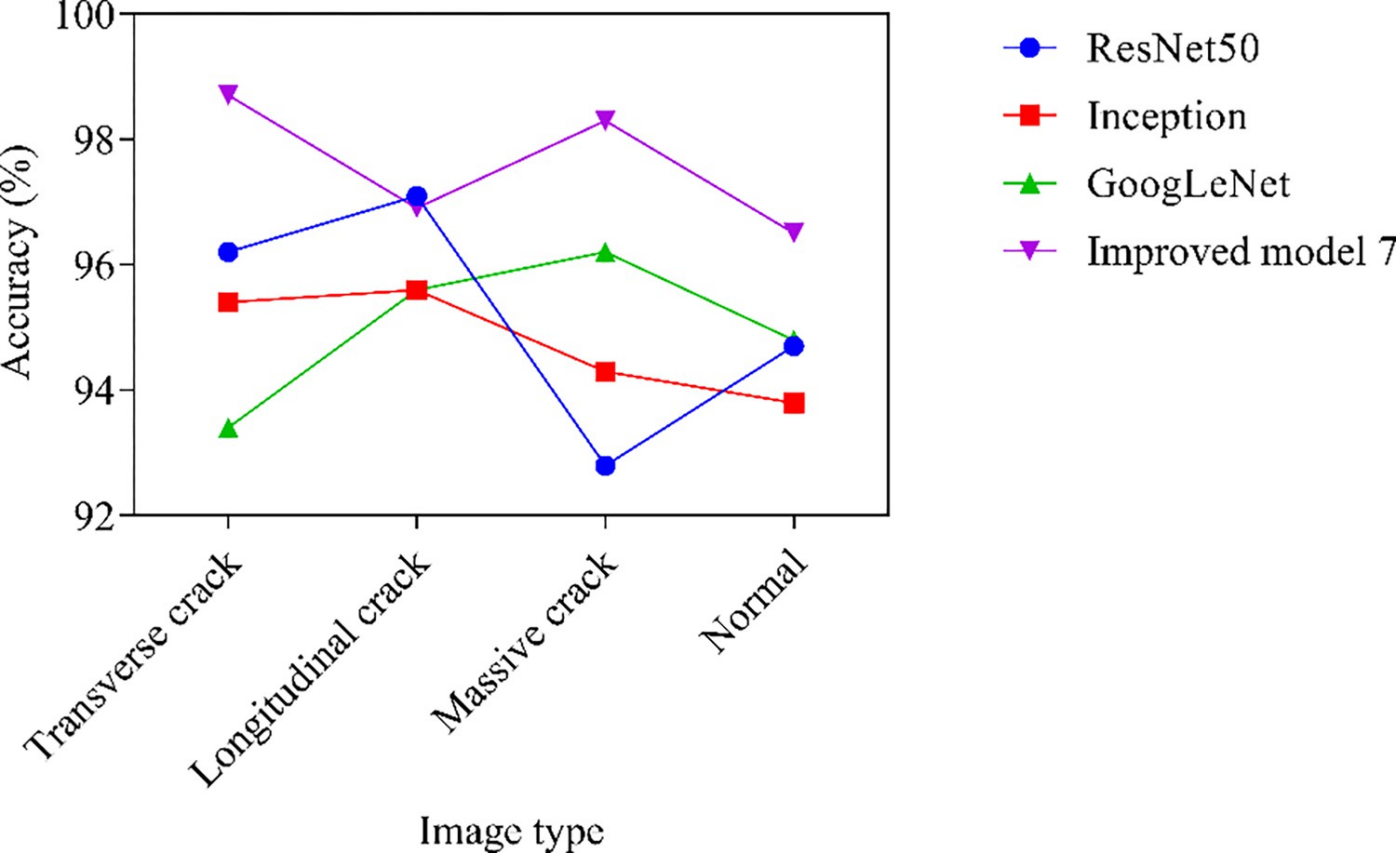
Recognition accuracy results.

The cracks on the reservoir panel are a potential safety hazard. Cracks may lead to reservoir leakage, structural weakening, and even catastrophic failure. Through crack image recognition technology, cracks can be regularly monitored and identified, and potential problems can be recognizing in a timely manner, ensuring the structural safety of the reservoir. Under normal light conditions, the recognition accuracy results are shown in Fig 6.

The Improved Model 7 has made three improvements simultaneously, with recognition accuracies of 98.7%, 96.9%, 98.3%, and 96.5% for transverse cracks, longitudinal cracks, massive cracks, and normal cracks images, respectively. The average recognition accuracy of ResNet50, Inception, GoogLeNet, and Improved model 7 are 95.2%, 94.8%, 95.0%, and 97.6%, respectively. By combining Leaky ReLU, attention mechanism, and residual connectivity, Improved Model 7 can more effectively extract and utilize features, enhancing the model’s recognition ability for complex crack morphology. These improvements not only enhance the model’s expressive power and robustness, but also enhance the stability during the training process, significantly improving the accuracy of crack recognition.

The recognition results are shown in Table 5.

**Table 5:** Recognition results.

Index	Accuracy (%)	Matthews correlation coefficient considers
ResNet50	95.2	0.95
Inception	94.8	0.94
GoogLeNet	95	0.94
EfficientNet	95.5	0.95
Transformer	95.3	0.96
Improved model 7	97.6	0.98

### 4.3 Results of Matthews correlation coefficient

**Fig 7:**
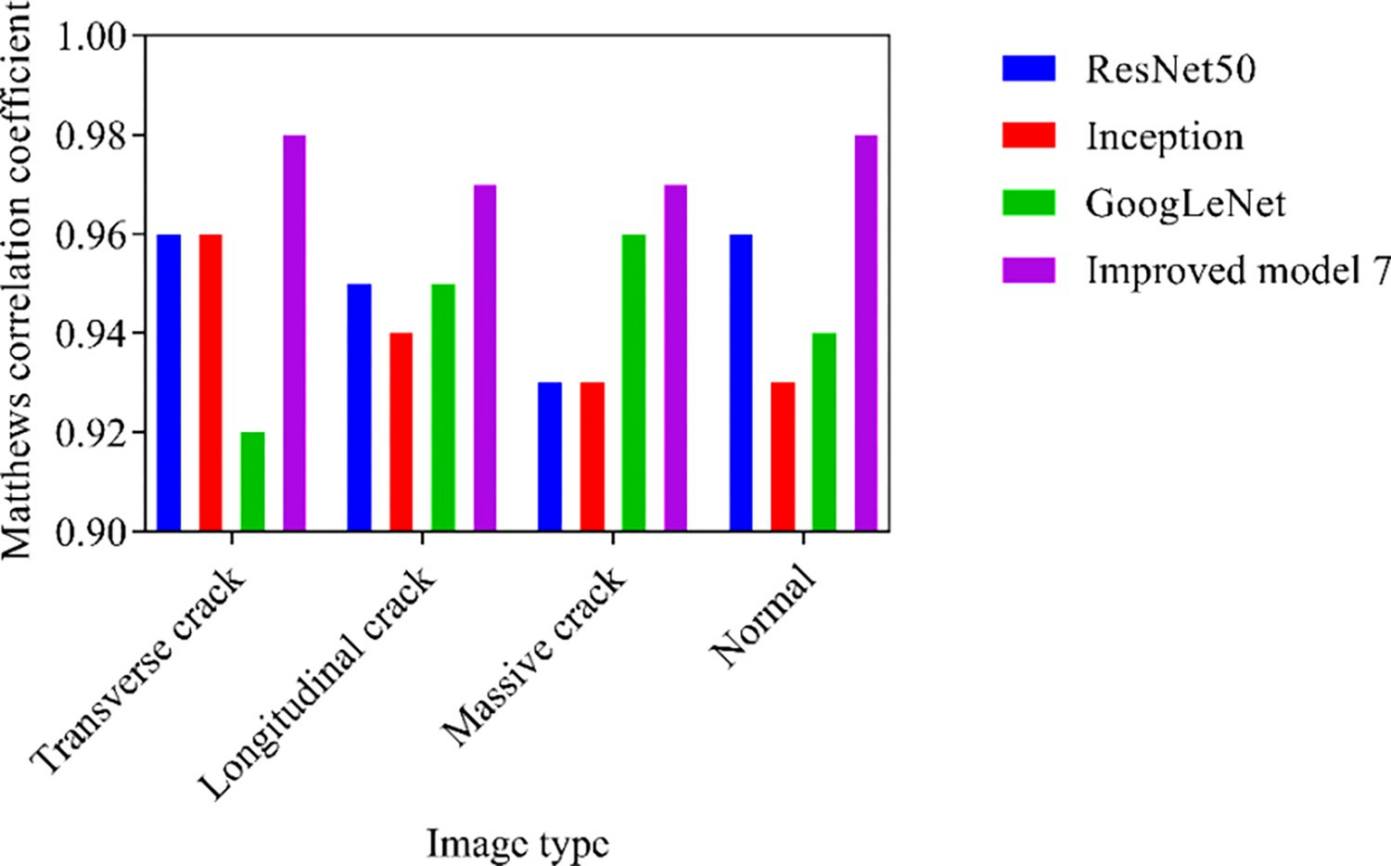
Results of Matthews correlation coefficient analysis.

Although accuracy can reflect the overall accuracy of model classification, the distribution of categories in the dataset images is not absolutely balanced. Under normal light conditions, the Matthews correlation coefficient analysis results are shown in Fig 7.

The Matthews correlation coefficient considers all elements in the confusion matrix, which can more comprehensively reflect the classification performance of the model. The recognition results of the Matthew correlation coefficient of Improved Model 7 are consistently higher than ResNet50, Inception, and GoogLeNet in the four types of images. The average Matthews correlation coefficients for ResNet50, Inception, GoogLeNet, and Improved model 7 are 0.95, 0.94, 0.94, and 0.98, respectively. The Improved Model 7 applies an attention mechanism that can be weighted based on the importance of feature maps, highlighting key features and suppressing irrelevant information. This enables the model to more accurately recognize and classify crack features when processing crack images. Moreover, Improved Model 7 optimizes the structure of residual connections, making information transmission smoother and reducing the problems of vanishing and exploding gradients. This helps with the training of deep networks, improving the stability and recognition accuracy of the model, especially under long-term training or deep network structures.

### 4.4 ROC results

**Fig 8:**
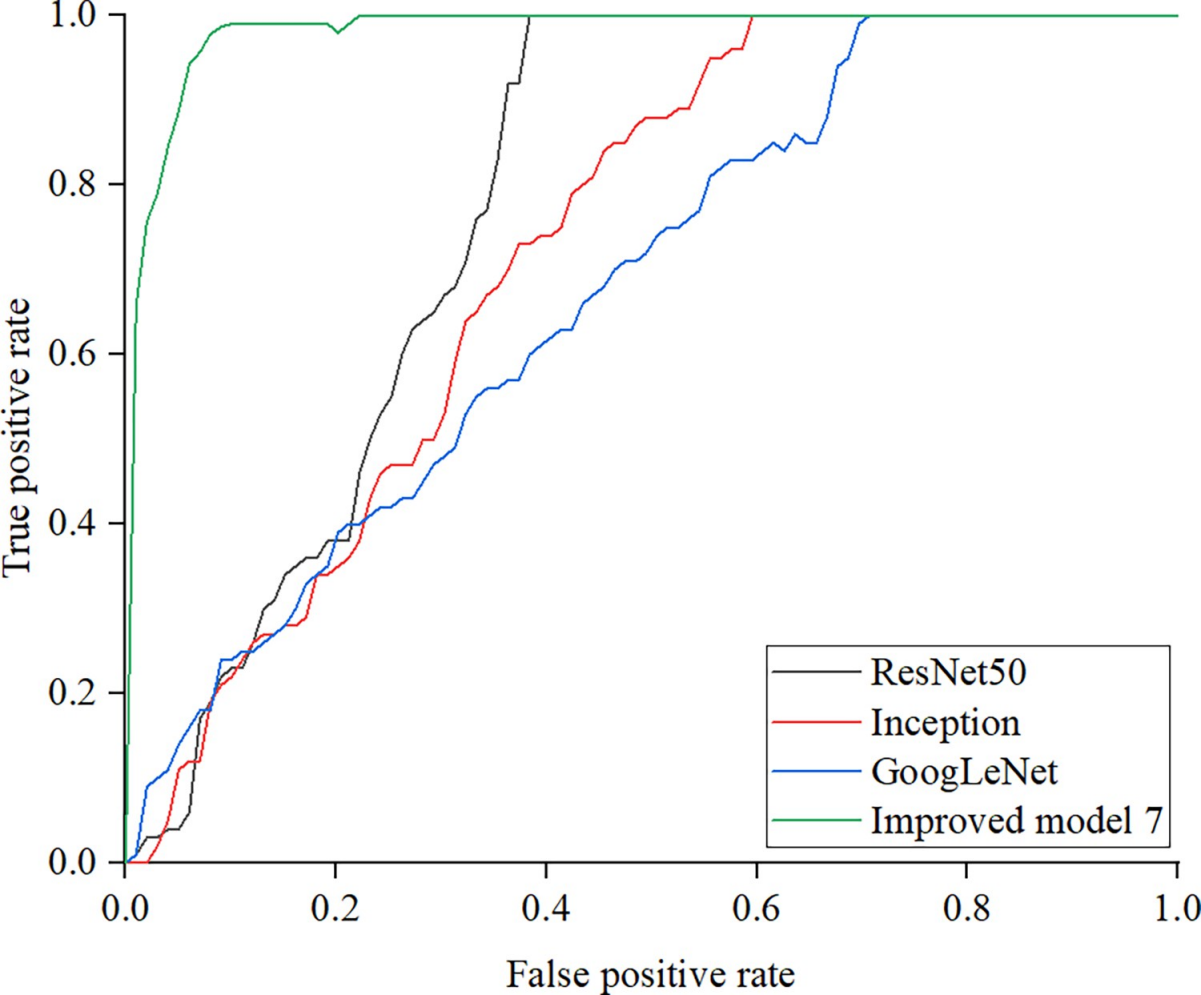
Comparison of ROC curves.

Under normal light conditions, the ROC curves of ResNet50, Inception, GoogLeNet, and Improved model 7 are compared as shown in Fig 8.

The diagonal of the ROC curve indicates that the model has no discriminative ability. From the development trend of the curve, it can be seen that the Improved Model 7 is closest to the upper left corner, and the area below the curve is the largest. This indicates that the Improved Model 7 performs better in image recognition of cracks in bituminous concrete reservoir panels than ResNet50, Inception, and GoogLeNet. The improved Xception model performs well in feature extraction, information transmission, and model training through depthwise separable convolution, Leaky ReLU activation function, application of attention mechanism, optimization of residual connections, and efficient structural design. These improvements enable the Xception model to more accurately recognize various types of cracks when processing crack images of bituminous concrete reservoir panels.

### 4.5 Stability results

**Fig 9:**
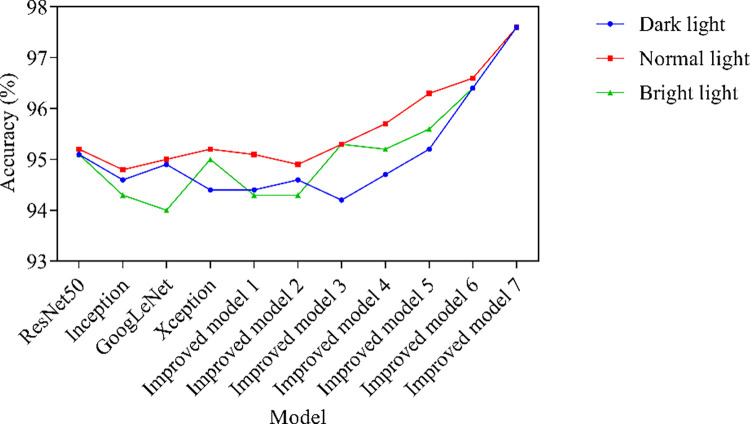
Stability analysis results.

The changes in lighting environment directly affect the quality of the image, including changes in brightness, contrast, color, and other aspects. For example, in low lighting environments, images may experience overexposure or heavy shadows, while in high lighting environments, images may experience issues such as loss of details or reflection. Therefore, analyzing the lighting environment can help evaluate the changes in image quality under different environments, and thus carry out targeted image enhancement or preprocessing to improve the stability of recognition algorithms. The stability analysis results are shown in Fig 9. Dark light refers to weak ambient light, resulting in insufficient image brightness and difficult to discern details. It usually manifests as a darker image with more noise. Quantitatively described as image brightness below 200, poor contrast, and possible blurring. Normal light refers to images under normal natural lighting conditions, with uniform lighting and clear details. Between 200–230 brightness, the image contrast is moderate and details are easy to discern. Bright light refers to strong ambient light, which may cause overexposure or reflection, and loss of details. Image brightness exceeds 230, and highlight areas may lose details or be overexposed.

Fig 9 shows the recognition accuracy results of 11 models in three lighting environments. The recognition accuracy in normal light is generally better than that in dark or bright light. This is because under normal light, the image quality is better, including brightness, contrast, color, etc., which are closer to the real scenario, and the details of cracks are clearer. In contrast, in environments with dark light, images may appear blurry and noisy, while in environments with bright light, images may experience overexposure or reflection, which can affect the accurate recognition of cracks by recognition algorithms. From the accuracy results of recognition, it can be seen that the Improved Model 7 always has the highest recognition accuracy in three lighting environments, with a recognition accuracy of 97.6%. However, the recognition accuracy of other models may fluctuate in different lighting environments. The stability performance of the Improved Model 7 in recognizing cracks in bituminous concrete reservoir panel images is superior to other models, mainly due to its use of depth separable convolutional structure, residual connection and attention mechanism, Leaky ReLU activation function, structural optimization and efficient design, and a series of technical means. This makes the model have better feature extraction ability, higher generalization ability, and stronger anti-interference ability, thereby improving the stability and reliability of the model in complex scenarios.

**Fig 10:**
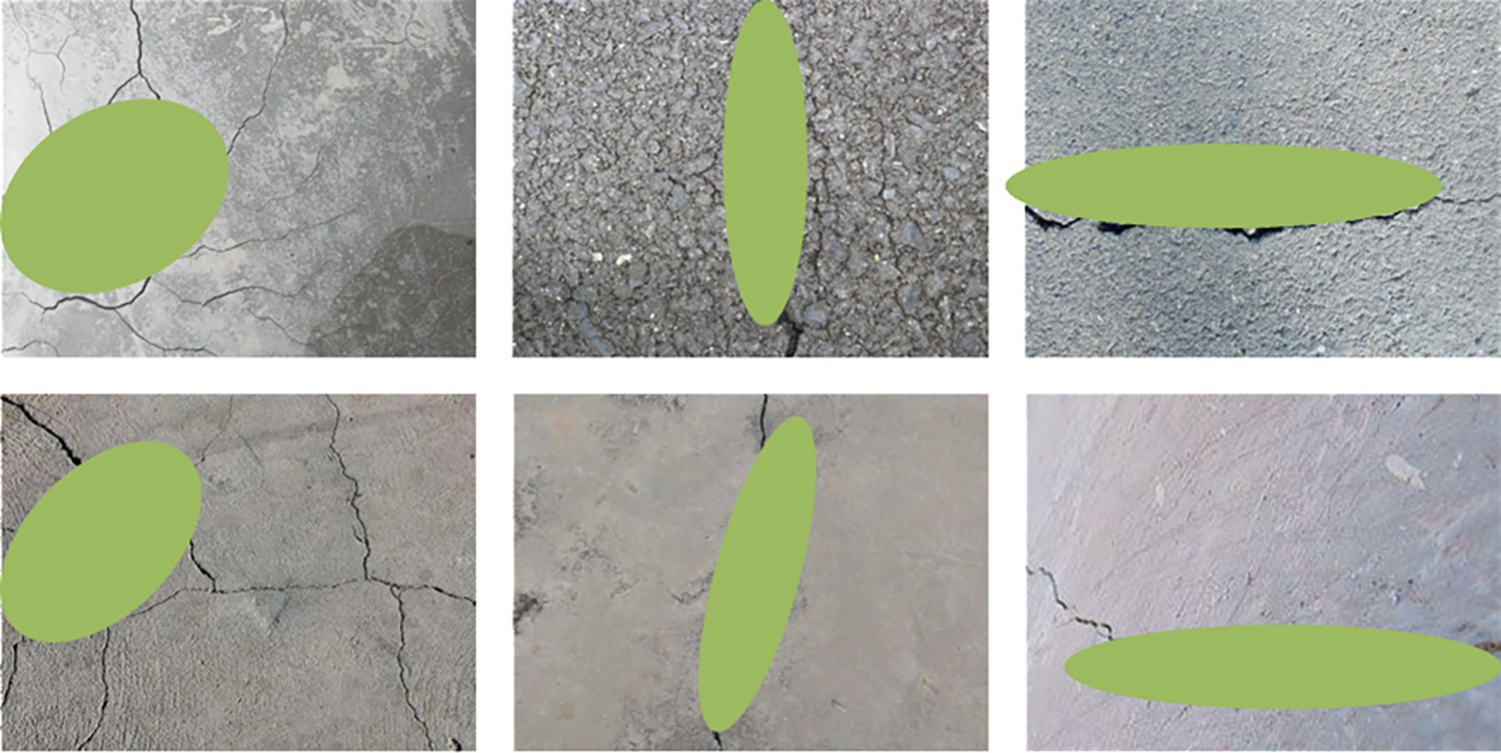
Crack detection results of the model in this article.

The crack detection results of the model in this article are shown in Fig 10.

## 5. Conclusions

The experiment compared the recognition performance of the model in this article with traditional ResNet50, Inception, and GoogLeNet models, as well as various improved Xceptions. The results showed that the Improved Model 7 in this article had higher accuracy in crack image recognition and could still maintain high stability in different lighting environments. The Improved Model 7 achieved a recognition accuracy of 97.6% in dark light, normal light, and bright light conditions. Based on the Xception model, the accuracy and stability of the recognition algorithm were effectively improved by applying techniques such as attention mechanism, residual connections, and Leaky ReLU activation function. By automatically recognizing cracks, it is possible to promptly detect and repair cracks in the reservoir panel, to improve the safety and stability of the reservoir, reduce the risk of accidents, and ensure the safety of people’s lives and property. However, there are still some shortcomings in this study. When collecting data, only the influence of the lighting environment on recognition results is considered, and the analysis of other environmental factors may also have an impact on recognition results. In the future, datasets can be expanded, and sample diversity and quantity can be increased, improving the adaptability and generalization ability of models. Future research can focus on further improving the accuracy and comprehensiveness of crack detection by estimating the length and width of cracks. Combining deep learning with image segmentation technology to accurately extract the geometric features of cracks will help quantify the severity of cracks and assess the health of road structures. In addition, exploring multimodal data fusion can enhance the robustness of the model in complex environments.
