# Correction: Image recognition technology for bituminous concrete reservoir panel cracks based on deep learning

**DOI:** 10.1371/journal.pone.0335901

**Published:** 2025-10-31

**Authors:** Kai Hu, Yang Ling, Jie Liu

There are errors in the author affiliations. The correct affiliations are as follows:

Kai HuID^1^, Yang Ling^2^, Jie Liu^3^

1 Institute of Civil and Architecture Engineering, Xi’an Technological University, Xi ‘an 710021, Shaanxi China, 2 Shaanxi Qinyuan Tendering Co., Ltd, Xi ’an, Shaanxi, China, 3 Shanxi Yuanqu Pumped Storage Co., Ltd, Yuncheng, Shanxi, China

In the Collection of crack image data subsection of the Materials and methods, there are errors in the third paragraph. The correct paragraph is: High-resolution digital cameras are used to capture detailed information of cracks in three types of lighting environments. The new dataset contains images from multiple geographic regions and environmental conditions, covering different seasonal changes and multiple types of reservoirs. The data was collected from June to December 2023 and captured using a high-resolution digital camera (Canon EOS 5D Mark IV) to ensure clear presentation of crack details. The image data comes from a dam in China. Due to potential conflicts of interest, the specific sampling locations mentioned in the article need to be removed, or replaced to prevent adverse effects on property owners. A variety of climate, light and environmental factors are taken into account, which helps to improve the universality and robustness of the crack detection model. Since the collected image data is not much, the crack image dataset in this paper was constructed by combining the images in the public dataset rdd2022. The collected image information is shown in Fig 1.
